# Frequency of Specific and Non-specific Inhibitors in Haemophilia A Patients

**DOI:** 10.7759/cureus.26008

**Published:** 2022-06-16

**Authors:** Javeria Ashfaq, Faryal Tariq, Rehana Ahmed, Warkha Thakur, Madiha Abid, Munira Borhany

**Affiliations:** 1 Clinical Haematology, National Institute of Blood Diseases and Bone Marrow Transplantation, Karachi, PAK; 2 Haematology, National Institute of Blood Diseases and Bone Marrow Transplantation, Karachi, PAK

**Keywords:** fviii, non specific inhibitors, plasma, factor viii inhibitors, factor viii, s: haemophilia complications

## Abstract

Objective: To determine the frequency of specific and non-specific inhibitors in haemophilia A patients.

Study design: This is a cross-sectional study.

Patients and methods: A total of 150 male haemophilia A patients were included in this cross-sectional study at the National Institute of Blood Diseases and Bone Marrow Transplant (NIBD), Karachi, Pakistan, from September 2019 to January 2022.

Results: Among 150 patients included in this study, 23 (15.3%) had an inhibitor and 127 (84.6%) did not. All patients had specific inhibitors against Factor VIII (FVIII). Non-specific inhibitors were not identified in our population. Among the patients in the inhibitor group, there were 13 (56.5%) in the severe (<1%) category. There were 10 (43.5%) patients in the moderate (1-5%) category. There were no patients in the mild category. The median inhibitor level was 15.4 Bethesda unit (BU).

Conclusion: The development of inhibitors has not been identified as a major problem in our population. However, it is noteworthy that only 15.3% of patients with haemophilia A developed inhibitors in this data set. They were essentially treated with plasma and its products.

## Introduction

Haemophilia A affects one in 5000 men. It is an X-linked hereditary disorder characterized by bleeding due to the lack of clotting factor VIII (FVIII). FVIII is a cofactor, which is responsible in the instigation of factor X via factor IXa. Classification of point mutations in the human FVIII gene has been rather challenging owing to its huge size and its increasingly complex design [1].

Subjects are noted to present themselves to the hospital with bleeding gums, epistaxis, hemarthrosis, and hematoma [2]. It is challenging to control incidents of bleeding among these patients. Literature shows that replacing FVIII is a reliable treatment option to halt bleeding [3]. However, the development of neutralizing antibodies against the administered exogenous FVIII, which are known as inhibitors, has been a longstanding complication [4].

Inhibitors are classified as specific and non-specific inhibitors (lupus). They are antibodies that are responsible for neutralizing factors that result in clotting. FVIII inhibitor results from alloantibodies among subjects with haemophilia A who have been given exogenous FVIII [5]. The development of antibodies is multifaceted, linking both non-genetic risk factors as well as endogenous genetic factors.

FVIII mutation has been researched widely. Scientists have demonstrated an increasing interest in the effect of polymorphisms when studying immune-regulatory genes. Approximately 20-30% of severe haemophilia A cases and 5-15% of moderate to mild haemophilia A cases tend to acquire inhibitors [4,5]. It has been indicated in research that huge deletions, impede mutations of codon and inversions 22 were correlated with bigger odds to make the inhibitor as opposed to missense mutations and minor deletions (5% vs 35% ). Exposure to certain commodities in the early part of life escalates the probability of its development [6].

Scientific progress has led to the manufacturing of plasma-derived, virally attenuated, coagulation factor products. There are recombinant FIX and FVIII distillates available as well. All these developments have resulted in the elimination of complications from severe bleeding, for instance, the danger of transmission of infections and haemophilic arthropathy. Literature has shown that one of the challenging complications of haemophilia therapy is the development of inhibitory antibodies [7]. The consequence of the development of inhibitors among haemophiliac patients is a very problematic hemostasis, particularly post elective surgery and through acute episodes of bleeding, consequently raising mortality and morbidity [7].

It is estimated that about 80% of the haemophiliacs are in developing countries, where receiving appropriate treatment is possible only for a few patients [4]. In Pakistan, it is noted that haemophilia management and treatment are inadequate. Due to the increased costs and unavailability of factor concentrates for use in our clinical setting, fresh frozen plasma (FFP) is frequently utilized as the therapeutic modality [4].

This study aims to determine the frequency of specific and non-specific inhibitors among haemophilia A patients.

## Materials and methods

A total of 150 haemophilia A male patients were included in this cross-sectional single-centre study at the National Institute of Blood Diseases and Bone Marrow Transplant (NIBD), Karachi, Pakistan, from September 2019 to January 2022. Patients who had bleeding disorders other than haemophilia A were excluded. Approval of the Institutional Review Board/Ethics Committee, Haematology, NIBD, Karachi, Pakistan, was taken prior to conducting the study (approval number NIBD/RD-199/10-2019). Informed consent was obtained from the parents of patients, following complete disclosure about the risks and benefits of the study. Strict International Council for Harmonisation of Technical Requirements for Pharmaceuticals for Human Use (ICH) good clinical practise guidelines were followed.

Thorough history comprising bleeding episodes, the start of signs and symptoms, treatment particulars, kind of factor concentrates, and length was noted in a questionnaire. Venous blood samples were gathered in tubes enclosing 0.109 M (3.2%) trisodium citrate in a proportion of nine parts blood to one part anticoagulant, which was then centrifuged immediately at 1200 G for 15 minutes. FVII inhibitor was assessed by utilizing activated partial thromboplastin time (APTT)-based method. Normal pooled plasma and subjects' plasma together in a 50:50 mix were incubated for a period of 120 minutes, at a temperature of 37°C [8]. A quantitative assay was done and Bethesda unit (BU) was noted for patients who had positive inhibitor screening. BU is expressed as the quantity of inhibitor that would eventually neutralize half of one unit of added FVIII in 120 minutes at a temperature of 37°C. FVIII inhibitors are noted to be essentially time-dependent and are labelled as low titre when they are fewer than 5 BU detected, though high titre implies that more than 5 BU [9]. In case a test sample contained no inhibitor, the FVIII activity in the test sample mixture was expected to be equivalent to the control, and the residual FVIII activity was expected to be 100%. If the residual FVIII activity in a sample was between 80-100%, it was considered that sample contained no inhibitor.

Statistical analysis

Data analysis was carried out on IBM SPSS Statistics for Windows, Version 23.0 (Released 2015; IBM Corp., Armonk, New York, United States). Descriptive analysis, i.e., frequency and percentage were computed for categorical variables of gender, family history, and factors, whereas mean and standard deviation were predictable for quantitative variables of age, age of diagnosis in years and inhibitor in BU. However, Chi-square was applied for categorical variables, i.e., specific and non-specific inhibitors.

## Results

**Figure 1:**
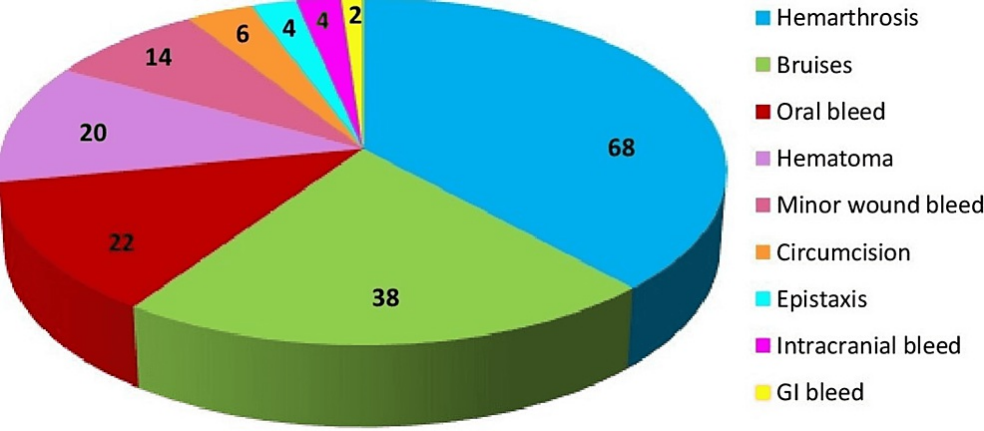
Frequency of bleeding in haemophilia A patients in the study

A total of 150 patients were included in this study. All of them (100%) were males. The median age at diagnosis was 0.8 years. Family history for haemophilia A was present in 60 (40%) patients. There was no family history of inhibitors in our patients. The frequency of bleeding type in our patients is shown in Figure 1. There were nine (6%) mild, 58 (38.7%) moderate, and 83 ( 55.3%) severe haemophilia A patients. There were 23 (15.3%) patients who had the inhibitor and 127 (84.6%) who did not. Table 1 shows the demographic characteristics of our patients.

**Table 1:** Demographic characteristics of patients in the study

Table 1: Demographic Characteristics (N=150)		
Age (Median (IQR))		
Years	15 (15.5)	
Age of diagnosis (Median (IQR))		
Years	0.8 (1.5)	
Gender (%)		
Male	150 (100)	
Family history of haemophilia A (%)		
Positive	60 (40)	
Factor (%)		
Mild	9 (6)	
Moderate	58 (38.7)	
Severe	83 (55.3)	
Inhibitor (%)		
Specific	23 (15.3)	
Non-specific	0	
Inhibitor Titre (Median(IQR))		
Bethesda unit		15.4 (31.2)

After stratifying the data in Inhibitor positive patients, the data showed that there were 13 (56.5%) patients in the severe (<1%) category, 10 (43.5%) patients in the moderate (1-5%) category, and no patients in the mild category. All patients had FVIII specific inhibitor and non-specific inhibitor (lupus antibody) was not identified in any patient. The results are shown in Table 2.

**Table 2:** Association of inhibitor with severity of haemophilia A Note: P-value > 0.05 found to be insignificant

Specific Inhibitor (BU)			P-value
Severity	Positive	Negative	0.407
Mild	-	9 (7.1%)	
Moderate	10 (43.5%)	48 (37.8%)	
Severe	13 (56.5%)	70 (55.1%)	

## Discussion

It is noteworthy that only 15.3% of the haemophilia A patients had developed inhibitors in this data set. They were essentially treated with plasma and its products. Haemophilia has a treatment cost that is excessive and, hence, its management is a burden for patients dwelling in developing countries. Preceding research regarding the range of congenital bleeding disorders has demonstrated that haemophilia A is found among the population of Pakistan [10]. Haemophilia is managed by using factor replacement therapy (FRT), which is primarily composed of FFP. Access to FFP is more conceivable for the Pakistani population. The challenges faced by patients in the developing world vary from a dearth of access to the suitable treatment and incorrect diagnosis owing to inadequate comprehension and lack of resources, resulting in an increased risk of morbidities among these subjects. Inhibitors are detected when a subject with haemophilia experiences an immune reaction towards clotting factor distillates. Allo-antibodies are present in about 20-30% of the haemophilia A subjects and are prevalent among those with severe disease as compared to moderate or mild categories [11]. Our study also found inhibitor development to be in a higher proportion among the severe category, that is 56.5%, as opposed to moderate and mild types and these results are close to a prior meta-analysis [12]. Further, results from Borhany et al. [4] have shown comparable results to our study.

Many factors are responsible for inhibitor development. For instance, the nature of gene defect, ethnicity, the severity of haemophilia A, intensive factor exposure when surgery takes place, on-demand treatment regimens, or prophylactic treatments. Also, literature shows that it matters what source of FVIII is used in the replacement therapy as it might have an influence on the development of the inhibitor [13]. However, Gouw et al. [14] had come across contradictory evidence in their study. They demonstrated that there was no significant distinction in the risk of emerging inhibitors among subjects who were getting FVIII. A literature review conducted by Iorio et al. showed that it was rather impossible to disprove or prove the theory that the use of rFVIII products is superior to that of pdFVIII and vice versa, in preventing the risk of developing inhibitors [15].

A Spanish research study demonstrated the cumulative incidence of inhibitors at different ages. According to the study, at three years of age, the incidence of inhibitors in patients of haemophilia A who had been treated with clotting factors was noted to be 41% before the age of six months, 29% between the age of six and 12 months, and 12% after one year [16]. A comparable inclination was detected in a study conducted in the Netherlands [17].

Clinically important FVIII inhibitors are typically detected when there is a lack of response to the replacement therapy. According to Borhany et al., about 15% of cases among a total of 21 haemophilia A cases were recognized with the help of inhibitors presence [4]. However, eight cases had a lower BU inhibitor titre and it was observed among 13 patients that there was a high BU. In that study, there was no family history of inhibitors [4]. Our study too did not show any family history of inhibitors in our patients. Literature has noted that the inhibitor formation pathogenesis and the cause that few inhibitors vanish is rather inadequately comprehended [18]. It has been noted that there is evidence suggesting that the development of inhibitory antibodies is a rather multifaceted activity linking together both genetic and non-genetic factors [19].

Limited resources and being a single-centre study were the major limitations of our study. Thus, further studies are needed in the future so that the multifaceted nature of the causes involved in inhibitor formation among the subjects may become more clear.

## Conclusions

The development of inhibitors has not been identified as a major problem in our population. However, it is noteworthy that only 15.3% of patients with haemophilia A developed inhibitors in this data set. Inhibitors are classified as specific and non-specific inhibitors, and non-specific inhibitors were not identified in our population.

Hemophilia A is mostly managed by FFP due to limited access to recombinant FVIII in developing countries. Our study population was essentially treated with plasma and its products.
